# Preliminary translational assessment of robotic surgery skills for vascular dissection: from simulator to in vivo porcine model

**DOI:** 10.1007/s11701-026-03662-5

**Published:** 2026-07-20

**Authors:** Alessandro Dario Mazzotta, Giulia Gamberini, Giuseppe Giuliani, Selene Tognarelli, Niccolò Petrucciani, Andrea Pichetto, Giancarlo D’Ambrosio, Gianluca Mennini, Andrea Coratti, Arianna Menciassi

**Affiliations:** 1 The BioRobotics Institute, Sant’Anna School of Advanced Studies, Pontedera (Pisa), Italy; 2 The Department of Excellence in Robotics & AI, Scuola Superiore Sant’Anna, Pisa, Italy; 3 Department of General Specialist Surgery and Anesthesiology, Sapienza University of Rome, Viale del Policlinico, 155 00185 Rome, Italy; 4 Health Science interdisciplinary center, Sant’Anna School of Advanced Studies, Pisa, Italy; 5 Department of General surgery, Misericordia Hospital, Grosseto, Italy; 6 Department of Medico-Surgical Sciences and Translation Medicine, Faculty of Medicine and Psychology, St Andrea Hospital, Sapienza University of Rome, Rome, Italy

**Keywords:** Robotic Surgical Procedures, Surgical Training Simulation, Learning Curve, Animal Models, Fellows, Robotic surgery

## Abstract

Robotic-assisted surgery (RAS) offers enhanced visualization, precision, and dexterity, but the absence of haptic feedback poses challenges during delicate dissection tasks such as vascular dissection. Simulation-based training has been proposed as a strategy to mitigate these limitations, yet evidence of translational effectiveness into in vivo surgical performance remains limited. We conducted a prospective, controlled feasibility study to evaluate the impact of a structured, simulator-based training program on robotic vascular dissection. Twelve novice surgeons were included in a prospective, controlled, non-randomized feasibility study. Six underwent structured dry-lab training with a sensorized high-fidelity vascular simulator, while six served as untrained controls, no baseline robotic performance assessment was performed before the intervention. Surgical performance was assessed during robotic vascular dissections in anesthetized porcine models using the da Vinci Xi platform. Performance was assessed by a single expert evaluator who was blinded to group allocation using the Global Evaluative Assessment of Robotic Skills (GEARS) and qualitative parameters including tissue handling, vessel exposure, and stapler placement. The trained group achieved significantly higher overall GEARS scores than the control group (25.7 ± 2.9 vs. 21.2 ± 2.4; *p* = 0.026). Depth perception was significantly improved in trained participants (4.33 ± 0.81 vs. 2.83 ± 0.75; *p* = 0.028). Trends toward enhanced bimanual dexterity and efficiency were observed but did not reach statistical significance. Qualitative analysis highlighted safer tissue handling, more consistent vessel exposure, and improved stapler positioning in the trained group compared with the controlgroup. Structured training with a sensorized high-fidelity vascular simulator was associated with better performance in selected components of robotic vascular dissection performance in an in vivo porcine model. These preliminary findings support the feasibility of this translational training pathway but require confirmation in larger randomized studies.

**Supplementary Information:**

The online version contains supplementary material available at 10.1007/s11701-026-03662-5.

## Introduction

Robotic-assisted surgery (RAS) has grown across various surgical specialties, offering several benefits such as enhanced visualization, precision, and dexterity for surgeons, as well as reduced postoperative length of stay, surgical infection and complications rates for patients [1–3]. Despite the technological advances, a critical limitation of widely adopted robotic platforms remains the lack of haptic feedback [4] especially with da Vinci Xi and da Vinci X Surgical System robots (Intuitive Inc., Sunnyvale, California, USA), which can compromise tissue handling and increase the risk of intraoperative complications, particularly during delicate tissue manipulation and vascular dissection [5–7]. In recent years, Intuitive Surgical introduced the da Vinci 5 system (Intuitive Surgical Inc., Sunnyvale, CA, USA), integrating force‑feedback (haptic) technology—a first among FDA (‑Food and Drug Administration) cleared devices in this class—which enables surgeons to sense push and pull forces, potentially improving tactile perception during delicate tasks. However, its clinical adoption remains limited, with broader deployment and long‑term outcome data still pending [8]. 

Vascular dissection is a key step in oncologic procedures such as lung lobectomy and liver resection [9, 10]. In this setting, vascular dissection requires fine manipulation and good instrument coordination to avoid excessive traction which can lead to severe adverse events such as vascular injury or bleeding [11] and to conversion to open surgery. The conversion rate due to bleeding in RAS generally ranges between 1% and 4%, but it varies depending on the specific procedure and its complexity [6, 7]. Studies have demonstrated that conversions to open surgery during RAS significantly worsen short-term outcomes, including higher complication rates (up to 31% Clavien-Dindo III–V, vs. 12%), longer hospital stays, and increased mortality (up to 21% vs. 4%) [12]. In general and colorectal surgical procedures, the conversion rate was 6.0%, with increased rates of surgical infections, cardiac complications, and reoperations [13].

RAS requires a different training approach compared to open or laparoscopic surgery, where surgeons directly touch tissues and can be mentored in a direct hands-on manner. To address these specific challenges and the complexity of vascular dissection procedures, simulation-based training has become an essential component of surgical education [14]. Indeed, simulated training provides trainees with the opportunity to learn RAS outside of the operating room, allowing novices to improve their skills without compromising patient safety [15]. However, robotic surgical training is inherently complex, requiring a combination of cognitive, psychomotor, and collaborative skills that do not directly transfer from laparoscopic or open surgery. These components highlight the difficulty of developing a uniform training curriculum, particularly given the increasing number of procedures performed using robotic systems [16]. Simulation-based training, specifically with physical simulators, represents a promising strategy to acquire ‘visual haptics’, i.e., the ability to understand deformation values through visual cues [17]. This is essential when dealing with delicate tissues such as blood vessels.

Building on our previously validated high-fidelity vascular simulator [18], we presented a sensorized high-fidelity physical vascular simulator for training in RAS. The system was preliminarly assessed in terms of face, content and construct validity, obtaining encouraging results [18]. Our simulator provides a reliable stratification of surgical experience within a in a simulated training setting [19]. However, in our previous work, the translation of skills acquired through simulation training into actual in vivo surgical performance was not investigated and requires further validation.

To provide insights regarding the translation of the acquired skills into in vivo surgical performance, we developed a pilot preclinical assessment protocol using a porcine model to assess whether training with our high-fidelity sensorized simulator results in measurable improvements in robotic surgical manipulation and performance.

We hypothesized that novice robotic surgeons undergoing structured training with a sensorized high-fidelity vascular simulator would demonstrate superior robotic vascular dissection performance during an in vivo porcine task compared with an untrained control group. Vascular dissection was selected as the translational target because it represents a high-risk surgical gesture in which precise tissue handling, depth perception, and force modulation are essential, particularly in the absence of direct haptic feedback. Moreover, it represents a surgical task commonly performed in different clinical specialties, whenever a target tissue or organ must be dissected or removed. The Global Evaluative Assessment of Robotic Skills (GEARS) was selected as the primary assessment tool because it is a validated instrument for evaluating core robotic surgical skills, including depth perception, bimanual dexterity, efficiency, force sensitivity, autonomy, and robotic control.

## Methods

### Study design

This was a prospective, controlled, non-randomized feasibility study evaluating the association between simulator-based training and robotic vascular dissection performance in an in vivo porcine model. Participants were voluntarily recruited among surgeons who were novices in robotic surgery and were pragmatically allocated in a 1:1 ratio to either the simulator-trained group or the untrained control group.

Allocation was established before the in vivo assessment and was not based on baseline performance, prior robotic exposure, or observed individual technical ability. No formal randomization or allocation concealment procedure was used.

The trained group underwent a structured training session using the sensorized high-fidelity vascular simulator, whereas the untrained group did not receive simulator-based training before the in vivo assessment. Both groups subsequently performed robotic-assisted vascular dissection in an in vivo porcine model using the da Vinci Xi surgical system.

No formal a priori sample-size calculation was performed because the study was designed as a preliminary feasibility assessment rather than a definitive efficacy trial. The sample size was determined by the exploratory nature of the protocol, the availability of live porcine models and the robotic platform, logistical constraints, and ethical considerations related to reducing animal use.

### Ethical and institutional approvals

The study was carried out within the framework of a formal collaboration between the Istituto di BioRobotica of the Scuola Superiore Sant’Anna (Pontedera (PI), Italy) and the Azienda USL Toscana Sud Est (Ospedale Misericordia di Grosseto, Italy), as established by institutional agreement no. 599, approved on June 11th, 2024. Training and validation activities were conducted at the Robotic Surgery School in Grosseto. All animal procedures were performed in accordance with institutional guidelines, under the supervision of qualified veterinary and surgical personnel, and in compliance with current European regulations on the use of animals for scientific and educational purposes (Directive 2010/63/EU) - protocol number 233/2009. All participants provided written informed consent for participation and anonymous data use.

### Participants

Twelve surgeons who were novices in robotic-assisted surgery were enrolled. All had previous experience in abdominal surgery but no hands-on robotic experience, thereby minimizing the potential influence of prior robotic exposure on performance. They were divided into two groups:

*Trained group* (*n* = 6): Participants who completed a structured dry-lab training program using our sensorized high-fidelity physical simulator.

*Untrained group* (*n* = 6): Participants who did not undergo simulator-based training before the in vivo procedure.

Baseline participant characteristics were collected to describe the cohort and assess comparability between groups, including level of surgical training, years of abdominal surgical experience, prior laparoscopic and open surgical exposure, previous simulation experience, and any previous exposure to a robotic console. Supplementary Table 1. No formal baseline robotic performance assessment was performed before allocation to the trained or untrained group.

### Simulator-based training in the dry lab

**Fig. 1:**
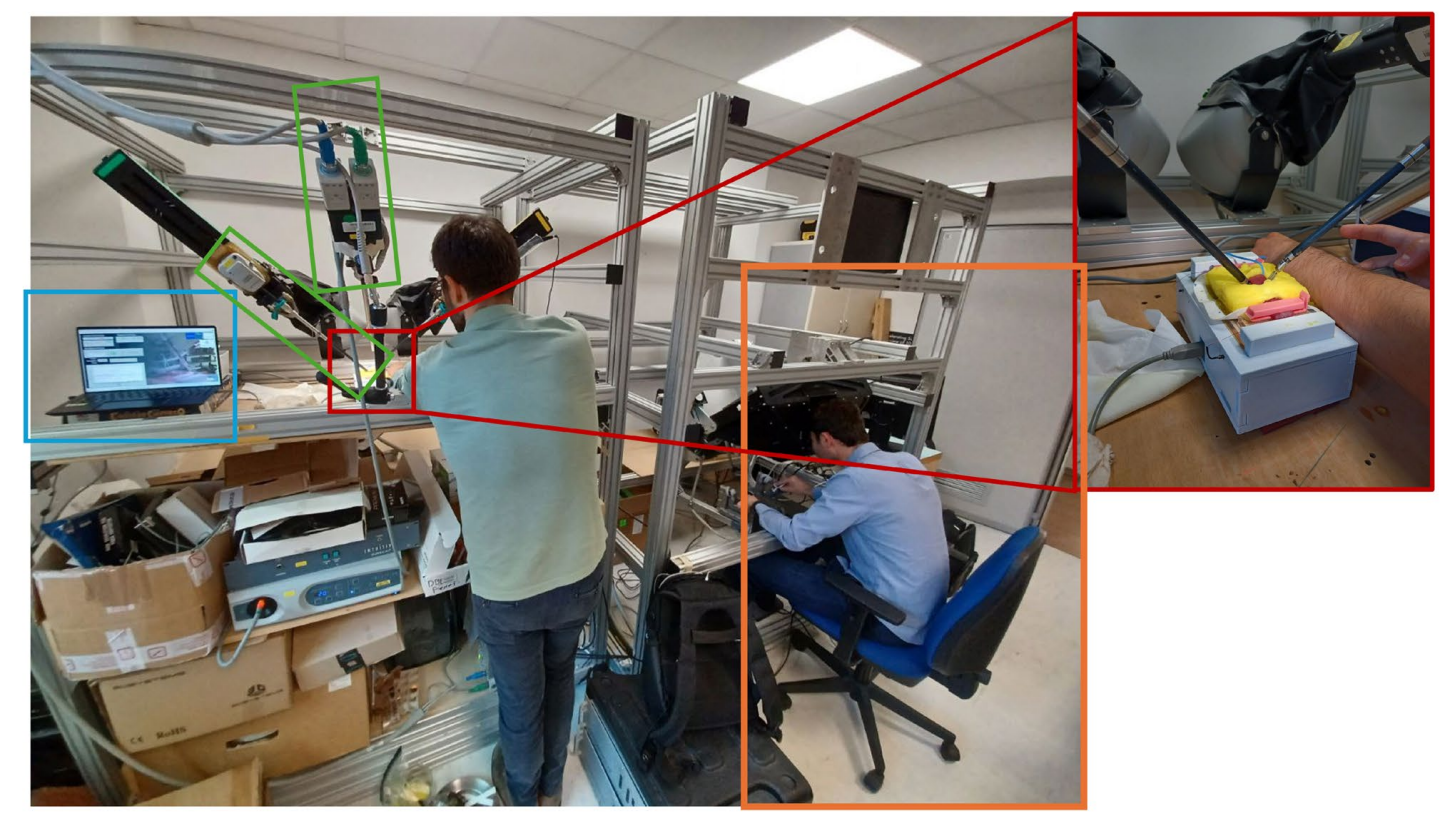
Dry-lab training setup in Pontedera using the dVRK platform and sensorized vascular simulator. The green boxes highlight the robotic arms, the blue box shows the computer-based sensor monitoring system, the red box shows the simulator, and the orange box highlights the surgeon in training.

Participants in the trained group underwent a structured dry lab training program at the BioRobotics Institute in Pontedera, using our sensorized high-fidelity physical simulator. The surgical task was performed using the da Vinci Research Kit (dVRK) platform [19, 20] Fig. 1. The high-fidelity physical simulator described in [18] was designed to mimic a vascular structure and ithe surrounding adipose tissue. The vessel was fabricated using silicone Ecoflex 00–30 (Smooth-On, USA), while the adipose tissue is realized using polyvinyl alcohol (PVA). The vessel was equipped with a customized fabric-based strain sensor to measure the deformation applied to the vessel during the surgical task. Moreover, to provide feedback to the users, three LEDs and a buzzer, placed on the simulator, can be activated when a when a predefined strain threshold is exceeded.

The training protocol included the following steps:

- **Video Tutorial**: Participants were first shown a standardized instructional video to introduce the principles of robotic vascular dissection and familiarize them with the key steps of the task. (Video 1)
- **Hands-on Familiarization**: Each participant completed a brief session to become familiar with the dVRK robotic console and the instrument setup.
- **Repetitive Simulation Task Execution**: Following familiarization, each participant performed five complete repetitions of the vascular dissection task:
  - Three with red-LEDs force feedback enabled.
  - Two without feedback to assess skill retention.

Sensor data (elongation signal) were acquired during each task repetition. Each participant in the trained group completed the simulation protocol only once. The protocol consisted of one standardized instructional video, one brief familiarization session, and five complete repetitions of the vascular dissection task, as described above. No additional unscheduled attempts were allowed. During the dry-lab training, participants performed the task individually, and the remaining participants were not allowed to observe the active operator at the console. This was done to minimize potential observational learning and to ensure that the measured improvement reflected direct simulator-based practice rather than learning from peer performance.

Simulated surgical procedures were performed using a dVRK platform. The standard operating configuration consisted of:

- Central view: 30° endoscopic camera positioned centrally.
- Left robotic arm: fenestrated grasper.
- Right robotic arm: scissors, with a vascular stapler available for use when required.

#### Data Acquisition

Sensor-derived metrics (elongation signal) were recorded during the sensor-enabled repetitions for further analysis.

### In vivo validation in the animal lab (Grosseto – robotic surgery school)

**Fig. 2:**
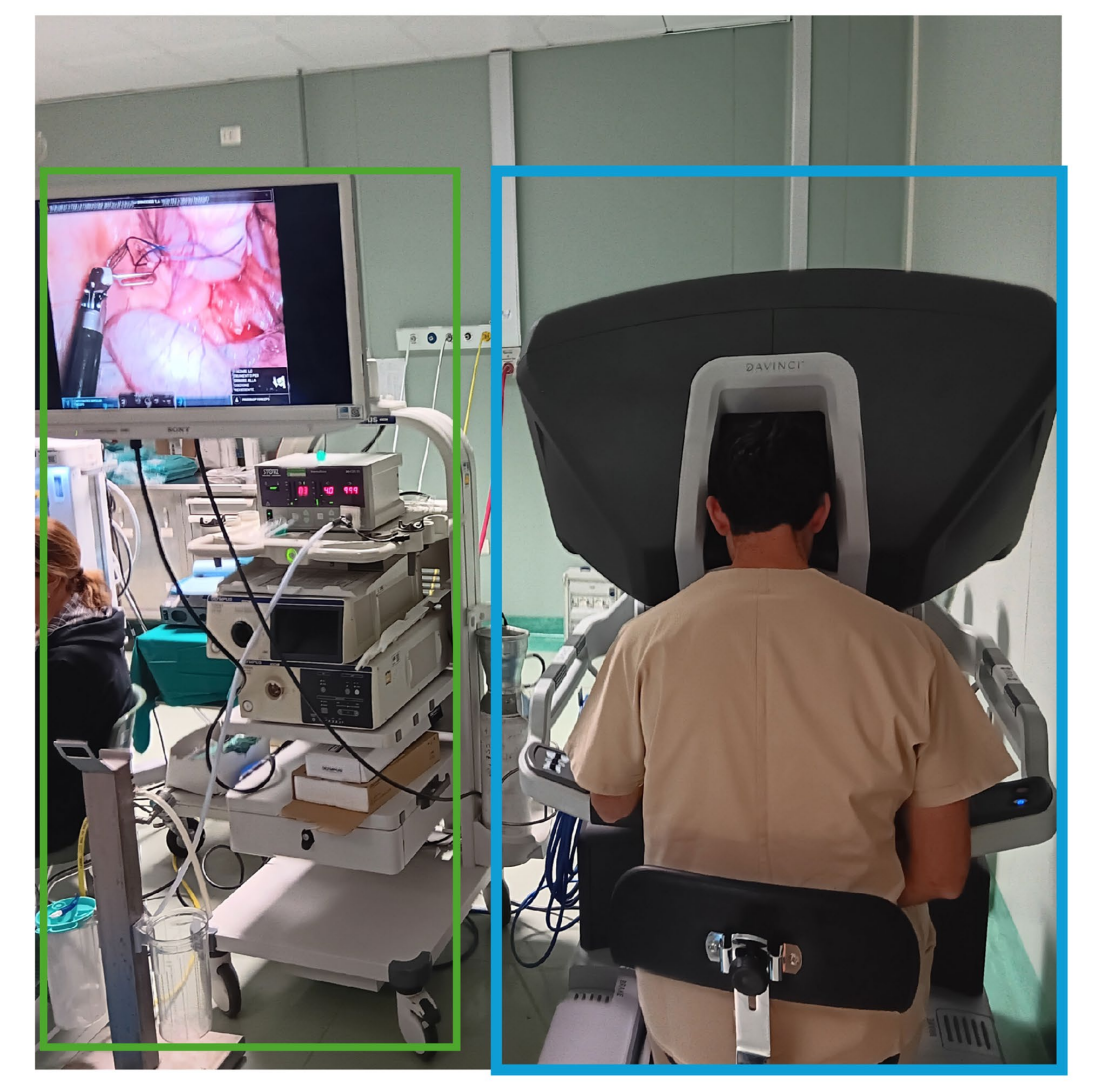
Animal-lab validation in Grosseto using the da Vinci Xi platform on a porcine model. The blu box highlights the surgeon operating at the robotic console.

The animal validation phase was conducted at the Robotic Surgery School in Grosseto, using live porcine models under general anesthesia (Fig. 2).

Surgical procedures were performed using a da Vinci Xi robotic surgical system, and the standard operating setup included:

- A 3D 30° endoscopic camera centrally positioned.
- A fenestrated grasper or Maryland bipolar forceps mounted on the left robotic arm.
- A scissor on the right robotic arm.
- A vascular stapler available for use on the right arm when required.

All 12 participants operated from the master console, with the surgical field displayed for the rest of the team in the surgical room on a high-definition monitor connected to the endoscopic camera. Each participant performed one in vivo robotic vascular dissection task at the master console. No participant was allowed to repeat the in vivo task or to perform additional console attempts before evaluation. To reduce the potential effect of observational learning, candidates who had not yet completed their assessment were not allowed to observe the console performance of other participants. The surgical field was displayed only for the operating team required for procedural safety and assistance.

The evaluation procedure involved vascular dissection tasks targeting:

- Iliac vessels.
- Gastroepiploic vessels.
- Portal vein branch or hepatic artery.

Because the porcine model presents inherent anatomical variability, vascular tasks were selected to reproduce comparable technical requirements rather than identical anatomical conditions. Across the different target vessels, the task consistently required circumferential vessel exposure, controlled dissection close to a vascular structure, safe passage around the vessel, and preparation for vascular stapling. The anatomical comparisons were decided in accordance with expert surgeons to provide a consistent task to the users across the two different groups. Nevertheless, differences in vessel depth, surrounding tissue, and accessibility may have influenced task difficulty and were considered when interpreting the results.

In order to optimize the use of resources, a single porcine model was utilized for multiple procedures. Whenever feasible, trained and untrained participants were assessed in an alternating temporal sequence rather than in separate consecutive blocks, in order to reduce systematic bias related to task order, progressive team familiarization, or procedural flow. However, the exact sequence was also influenced by logistical constraints related to animal model availability, operating room organization, and procedural safety. Specifically, each animal was assigned to three or four operators: two were assessed on lower gastrointestinal procedures, and two on upper gastrointestinal procedures. This approach, although logistically challenging, was necessary due to the limited availability of animal models and ensured maximal utilization while adhering to ethical principles for the reduction of animal use in research.

An experienced robotic assistant supported the procedures but did not directly intervene in the dissection in the dissection. All procedures were video recorded using an external camera that captured the console screen.

### Expert performance evaluation

Video assessment was performed by a single blinded expert robotic surgeon with more than 10 years of experience in minimally invasive and robotic surgery using the GEARS framework. The reviewer was blinded to group allocation and evaluated all videos using the same standardized scoring framework. Videos were anonymized before evaluation and did not include participant names or explicit group identifiers. The evaluator was blinded to group allocation. However, the videos were not edited to alter procedural style, instrument movements, or task progression.

The evaluation was based on the following metric:

- **GEARS** (Global Evaluative Assessment of Robotic Skills) [21], which assesses six domains: depth perception, bimanual dexterity, efficiency, force control, autonomy, and robot control. (see Supplementary file 1).

In addition to the GEARS criteria, the following qualitative parameters were also evaluated:

- Exposure quality of the right and left vessel margins: adequacy of vessel visualization and control before stapling.
- Instrument-tip contacts with the vessel: unintentional contact between unintentional contact between the instrument tip and the vessel.
- Frequency of grasp-release movements: number of repeated clamping/unclamping and aberrant movements.
- Evidence of excessive vessel tension or friction: signs of overstretching or dragging of the vessel.
- Correct insertion and visualization of the stapler: whether stapler was inserted under direct visualization of the jaws around the vessel.

A structured qualitative assessment form was used to guide the reviewer’s comments and ensure consistency across videos. The qualitative assessment was descriptive, task-specific, and non-validated. For each case, the reviewer recorded whether vessel exposure was adequate, whether unsafe instrument–vessel contacts were observed, whether excessive traction or friction occurred, and whether stapler insertion was performed under direct visualization. These observations were used to support the interpretation of the GEARS domains, particularly force sensitivity, efficiency, and depth perception. The overall GEARS score was used as the primary quantitative outcome. The qualitative parameters did not contribute numerically to the GEARS score and were not integrated into the final 0–30 score. These observations were not treated as independent quantitative outcomes and were not used to modify the GEARS score. Instead, they were used as descriptive adjuncts to provide task-specific interpretation of tissue handling, vessel exposure, instrument–vessel interaction, and stapler positioning during robotic vascular dissection. Each video was assessed with a numerical GEARS score and accompanied by descriptive comments to support interpretation of the GEARS assessment.

No validated competency threshold has been established for GEARS assessment of robotic vascular dissection in an in vivo porcine model. Therefore, GEARS scores were interpreted as comparative performance measures between groups rather than as indicators of whether participants had achieved independent clinical competence.

### Statistical analysis

Continuous variables were summarized as mean ± standard deviation. Comparisons between the simulator-trained and untrained groups were performed using the Wilcoxon rank-sum test because of the small sample size and the exploratory nature of the study (a p-value < 0.05 was considered statistically significant) [22]. Statistical analyses were performed using MATLAB software (MathWorks Inc., Natick, MA, USA).

The primary outcome was the overall GEARS score, calculated as the sum of the six GEARS domains, with a total score ranging from 0 to 30. The individual GEARS domain scores were considered secondary exploratory outcomes. Qualitative task-specific observations related to tissue handling, vessel exposure, instrument–vessel contact, excessive traction or friction, and stapler positioning were analyzed descriptively and were not included in the inferential statistical analysis.

For the overall GEARS score and each individual GEARS domain, between-group mean differences were calculated and reported with 95% confidence intervals. Standardized effect sizes were calculated as Hedges’ g and reported with 95% confidence intervals. Hedges’ g was selected because it incorporates a correction for small-sample bias. Positive mean differences and positive Hedges’ g values indicated higher scores in the simulator-trained group.

No adjustment for multiple comparisons was applied to the individual GEARS domain analyses; therefore, these findings should be interpreted as exploratory.

Given the small sample size, effect sizes and 95% confidence intervals were calculated for the overall GEARS score and GEARS subdomains to improve interpretability of the magnitude and precision of the observed differences.

## Results

A total of 12 surgeons who were novices in robotic surgery participated in the animal validation phase: six in the trained group and six in the untrained control group. Regarding the primary outcome, the trained group had significantly higher overall GEARS scores than the untrained group (25.7 ± 2.9 vs. 21.2 ± 2.4, *p* = 0.026). The individual and summary scores are reported in Table 1.

**Table 1:** Overall GEARS score according to training group and anatomical target

Case Control	Trained = 6		Not Trained = 6		*P* Value
	Anatomy	Overall GEARS score	Anatomy	Overall GEARS score	
1	Right iliac vessel	27	Left iliac vessel	18	
2	Right iliac vessel	27	Left iliac vessel	21	
3	Gastroepiploic vessels	27	Gastroepiploic vessels	24	
4	Gastroepiploic vessels	28	Left portal vein	24	
5	Right iliac vessel	25	Left iliac vessel	20	
6	Gastroepiploic vessel	20	Hepatic artery	20	
Mean ± SD		**25.7 ± 2.9**		**21.2 ± 2.4**	**0.026**
Mean difference, 95% CI		**4.5 points**		**95% CI 1.0 to 8.0**	
Effect size		**Hedges’ g = 1.55**		**95% CI 0.23 to 2.87**	

Mean GEARS subdomain scores were numerically higher in the trained group across all evaluated domains Notably, depth perception was significantly better in trained participants (mean score 4.33 ± 0.81 vs. 2.83 ± 0.75, *p* = 0.028). Bimanual dexterity and efficiency also showed higher mean values in the trained group (4.33 ± 0.81 vs. 3.33 ± 0.51 and 4.33 ± 0.81 vs. 3.33 ± 0.81, respectively), although these differences did not reach statistical significance (*p* = 0.080 and *p* = 0.119). Force control, autonomy, and robot control showed no statistically significant between-group differences (Table 2).

**Table 2:** GEARS subdomain scores in trained and non-trained participants

GEARS subdomain	Trained participants						Trained mean ± SD	Non-trained participants						Non-trained mean ± SD	Mean difference, 95% CI	*p*-value
	T1	T2	T3	T4	T5	T6		U1	U2	U3	U4	U5	U6			
Depth perception	5	4	5	5	4	3	**4.3 ± 0.8**	2	2	3	4	3	3	**2.8 ± 0.8**	**1.5; 95% CI 0.5 to 2.5**	**0.028**
Bimanual dexterity	4	5	5	5	4	3	**4.3 ± 0.8**	3	3	4	4	3	3	**3.3 ± 0.5**	**1.0; 95% CI 0.1 to 1.9**	**0.080**
Efficiency	4	5	5	5	4	3	**4.3 ± 0.8**	2	4	4	4	3	3	**3.3 ± 0.8**	**1.0; 95% CI − 0.1 to 2.1**	**0.119**
Force control	5	4	4	4	4	4	**4.2 ± 0.4**	3	4	4	4	3	3	**3.5 ± 0.5**	**0.7; 95% CI 0.0 to 1.3**	**0.181**
Autonomy	5	4	4	4	4	3	**4.0 ± 0.6**	5	3	4	4	4	3	**3.8 ± 0.8**	**0.2; 95% CI − 0.7 to 1.1**	**0.863**
Robot control	4	5	4	5	5	4	**4.5 ± 0.5**	3	5	3	4	4	5	**4.0 ± 0.9**	**0.5; 95% CI − 0.5 to 1.5**	**0.448**

GEARS, Global Evaluative Assessment of Robotic Skills; CI, confidence interval; SD, standard deviation.

Values are reported as individual participant scores and mean ± SD. Mean difference refers to trained minus non-trained participants. Hedges’ g was reported as standardized effect size to account for the small sample size. Confidence intervals are reported to describe the precision of the estimated between-group differences

## Discussion

RAS is being adopted across several surgical specialties, and it is used for complex procedures involving delicate anatomical structures; given the lack of tactile feedback in RAS, training in RAS may benefit from simulation-based programs that enable novice surgeons to develop technical skills in a safe environment. In this pilot preclinical study, structured simulator-based training was associated with higher robotic vascular dissection performance scores in an in vivo porcine model, as reflected by a higher overall GEARS score and improved depth perception in trained participants. These findings suggest that selected skills acquired in a sensorized dry-lab environment may transfer to a higher-fidelity biological model. However, the results should be interpreted as preliminary and hypothesis-generating, given the small sample size, absence of baseline robotic performance testing, single-rater assessment, and lack of validation in clinical operating-room performance.

Participants who completed the dry-lab training program achieved significantly higher overall performance scores compared to their untrained counterparts (25.7 ± 2.9 vs. 21.2 ± 2.4; *p* = 0.026). Among the GEARS subdomains, depth perception showed a statistically significant improvement in the trained group (*p* = 0.028), while bimanual dexterity and efficiency demonstrated positive trends that did not reach statistical significance. These findings suggest that targeted preclinical training may be associated with measurable differences in performance, even in novice robotic surgeons.

Our blinded evaluation incorporated task-specific criteria derived from critical intraoperative observations. This led to the development of a task-specific descriptive assessment. From a task-specific perspective, trained participants more frequently demonstrated controlled tissue traction, progressive vessel exposure, and more consistent maintenance of the dissection plane. In contrast, untrained participants more often showed abrupt grasp-release movements, less stable instrument positioning, and greater difficulty maintaining adequate visualization of the vessel margins before stapler placement. These qualitative observations should be interpreted cautiously because the task-specific assessment form was not externally validated and was used only as a descriptive adjunct to the GEARS evaluation.

**Fig. 3:**
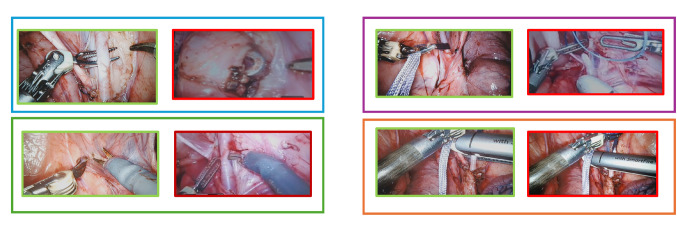
Examples of safe and unsafe gestures during robotic vascular dissection. Green boxes show controlled maneuvers; red boxes show inappropriate traction, exposure, or stapler positioning.

Attention was given to unsafe or inappropriate gestures (Fig. 3). These behaviors were considered potentially unsafe because they may reduce precision and increase the risk of tissue injury. The adaptation of the scoring system highlights the need for domain-specific metrics when evaluating robotic vascular dissection, where control, finesse, and respect for tissue are of paramount importance.

Another important point is that structured dry-lab training enabled participants to internalize a reproducible, stepwise approach to robotic vascular dissection, including vessel isolation, safe passage of the vessel loop, and accurate stapler positioning. This level of procedural schematization provides a basis for the standardization of complex technical gestures, which is essential in robotic surgery. In clinical settings, such structured techniques are potentially relevant to critical phases of hepatobiliary procedures, such as dissection of the hepatic hilum during liver resections, or the vascular control of short gastric vessels in gastrectomy. Our approach promotes a “step-by-step” procedural model, which may facilitate effective surgical learning [23]. This modularization facilitates the internalization of safe and reproducible techniques, allowing novice surgeons to progress systematically along the learning curve. In RAS, where intuitive gestures are limited by the interface and haptic feedback is absent, such structured learning pathways are essential for developing both technical proficiency and procedural confidence.

The concept of ‘visual haptics’ – interpreting tissue behavior through image-based feedback – is particularly relevant and may be developed in a simulated environment [17]. In other words, surgeons must learn to interpret changes in tissue deformation, color, and tension as proxies for tactile cues. This skill allows the operator to estimate applied forces, recognize imminent tissue failure, and guide precise dissection without direct tactile input. Our findings suggest that these competencies can be effectively cultivated in a simulated environment, where repetitive, targeted training accelerates the integration of visual and motor information, potentially supporting safer and more controlled performance in subsequent training environments.

In our study the transfer of skills from the simulator to the in vivo setting was preliminarly assessed and yielded preliminary evidence of between-group differences. Trained participants showed superior control during vascular manipulation, especially under conditions of vessel tension or when spatial constraints limited instrument triangulation. Our findings suggest that structured dry-lab simulator-based training may support the development of technical skills relevant to complex robotic dissection tasks, including those involving the splenic hilum, liver resections, and oncologic lymphadenectomy. The splenic hilum—a narrow anatomical space with high-risk vasculature—has been reported as technically challenging, with increased spleen volume and hilar hemorrhage identified as predictors of conversion to open or laparoscopic surgery [24]. Robot-assisted lymphadenectomy can pose considerable technical challenges. Managing major vessel lesions is challenging in the confinated pelvic space, and conversion is often required due to limitations such as poorly articulated instrument tips, counterintuitive movements, limited dexterity, and reduced depth perception [25, 26]. In this regard, our simulator can simulate lymphatic tissue, a critical component of staging and therapeutic procedures in surgical oncology. While previous models, such as the one developed by Kiely et al., have successfully demonstrated low-cost pelvic lymphadenectomy simulations using gelatin, cotton balls, and rubber tubing to replicate anatomical structures [27], our sensorized high-fidelity physical simulator offers additional features tailored specifically to robotic surgical training. Notably, our model integrates objective feedback provided through sensorization of the vascular structure, vessel deformation tracking, and standardized tasks with predefined performance endpoints, thereby enabling both formative assessment and procedural standardization. This expands the scope of simulation beyond anatomical dissection to include instrument-tissue interaction control, safety awareness, and team communication around critical steps, such as vascular stapling or managing vessel tension.

Using PVA to mimic lymphatic tissue, our model supports realistic dissection of external, internal iliac, and obturator lymph nodes—mirroring the anatomical complexity encountered during pelvic lymphadenectomy. In addition, our model supports realistic dissection of vascular structures similar to those encountered during robot-assisted radical hysterectomy or systematic nodal dissection during robotic-assisted radical hysterectomy or systematic nodal clearance.

Several studies have been carried out to validate simulation approaches on animal models. The work of Whitehurst et al. represented one of the first systematic attempts to measure the translation of skills from a simulation model to a real-world context: in that case, the use of an audiovisual feedback system improved performance in robotic urological surgery, resulting in shorter operative times and a faster learning curve and a faster learning curve [28]. Another study [29] analyzed the effectiveness of simulated robotic training, based on virtual suturing, in novices. Participants were divided into two groups: one underwent comprehensive training on a VR (Virtual Reality) simulator (Mimic dV-Trainer) until they achieved proficiency, and the other received simple orientation. Both groups were then evaluated in suturing on an in vivo porcine model. Surprisingly, no significant differences emerged in either time or GEARS scores, raising doubts about the real effectiveness of virtual training alone for technically complex tasks in biological environments. This study should be interpreted in light of several limitations.

As mentioned, although procedural standardization was pursued, potential residual learning effects related to observation, task order, and team familiarization cannot be completely excluded. To minimize this bias, participants were allowed only one in vivo console attempt, unscheduled repetitions were not permitted, and candidates who had not yet completed the assessment were not allowed to observe other participants performing the task. Nevertheless, given the logistical constraints of an in vivo porcine model, the order of assessment could not be fully randomized. The absence of formal randomization and allocation represents a methodological limitation. Although allocation was not based on prior robotic experience or observed technical ability, selection bias and baseline imbalance cannot be excluded.

The absence of baseline robotic skill assessment is an important limitation. Although all participants were novices in robotic surgery, we cannot exclude pre-existing differences in technical aptitude, visuospatial ability, laparoscopic experience, or familiarity with simulation environments.

Another limitation is the mismatch between the dry-lab and in vivo robotic platforms. Dry-lab simulation was performed using the dVRK platform with a fixed 2D camera, whereas the in vivo animal procedures were conducted using the da Vinci Xi platform with a 3D endoscopic view. This discrepancy may have influenced ergonomics, instrument handling, visual perception, and particularly depth perception. Therefore, the observed improvement in depth perception should be interpreted cautiously, as it may reflect both simulator-based training and adaptation to the 3D in vivo platform.

We acknowledge that the small sample size represents a limitation of this study. The inclusion of 12 participants could in principle limit statistical power, increase uncertainty around effect estimates, and restrict the generalizability of the findings. However, the present work was conceived as a preliminary preclinical feasibility assessment rather than a powered high sample size efficacy trial. The sample size was determined by the exploratory nature of the protocol, the use of a live porcine models, robotic platform availability, veterinary supervision, logistical constraints, and ethical considerations related to animal use. Small cohorts have also been used in previous early translational robotic simulation studies involving biological or porcine models to investigate feasibility, procedural transfer, or model validity [30, 31]. 

However, this methodological context does not eliminate the limitations associated with the small sample size of the present study. Therefore, the observed differences should be regarded as preliminary signals rather than definitive evidence of training efficacy.

The results should consequently be interpreted as exploratory and hypothesis-generating. Larger studies based on an a priori sample-size calculation, formal randomization, standardized anatomical tasks, multiple independent evaluators, and assessment of skill retention will be required to confirm the magnitude, reproducibility, and translational relevance of the observed effects.

The in vivo tasks were not anatomically identical, as vascular targets included iliac vessels, gastroepiploic vessels, and portal vein branch or hepatic artery targets. This heterogeneity may have introduced variability in task difficulty related to vessel depth, surrounding tissue, accessibility, and local anatomy. Although all tasks were selected to reproduce the same technical requirements of robotic vascular dissection in accordance with expert surgeons, this anatomical variability remains an important limitation and may have influenced the observed performance differences.

Another limitation was the involvement of a single blinded expert. Although blinding reduced the risk of assessment bias, inter-rater reliability could not be evaluated. Future studies should include multiple independent blinded raters and assessment of inter-rater agreement. The present study evaluates transfer from a sensorized dry-lab simulator to an in vivo porcine model and should not be interpreted as evidence of direct clinical transfer to the operating room. The porcine model represents an intermediate preclinical translational step, but clinical operating-room performance, patient safety outcomes, and long-term skill retention were not assessed in the present study. Recent literature confirms the increasingly central role of simulation as a tool for improving the safety, autonomy, and technical skills of trainee surgeons, but also highlights important methodological differences compared to our approach.

## Conclusion

This preliminary feasibility study suggests that structured training with a sensorized high-fidelity vascular simulator may be associated with better performance in selected aspects of robotic vascular dissection performance in an in vivo porcine model. The observed improvement in overall GEARS score and depth perception supports the potential translational value of simulator-based preparation for robotic surgery. Larger randomized studies incorporating baseline skill assessment, multiple independent raters, retention testing, and clinical validation are required before definitive conclusions can be drawn.

## Supplementary Information

Below is the link to the electronic supplementary material.

Supplementary Material 1

Supplementary Material 2

Supplementary Material 3

## Data Availability

No datasets were generated or analysed during the current study.
