# Supplementary material for: Preliminary translational assessment of robotic surgery skills for vascular dissection: from simulator to in vivo porcine model
Source: J Robot Surg. 2026 Jul 20;20(1):730. doi: 10.1007/s11701-026-03662-5 (PMC13385068; doi:10.1007/s11701-026-03662-5)
Supplement: Supplementary file 2 — Supplementary Material 2 [file 11701_2026_3662_MOESM2_ESM.docx]

Supplementary Table 1. Baseline characteristics of the two groups.

| **Characteristic** | **Trained (n=6)** | **Non-trained (n=6)** |
| --- | --- | --- |
| **Age (years)** |  |  |
| Mean ± SD | 35.8 ± 8.8 | 32.0 ± 4.1 |
| **Sex, n (%)** |  |  |
| Male | 3 (50.0%) | 4 (66.7%) |
| Female | 3 (50.0%) | 2 (33.3%) |
| **Surgical background, n (%)** |  |  |
| General Surgery | 6 (100%) | 6 (100%) |
| **Experience level, n (%)** |  |  |
| Resident in Surgery | 2 (33.3%) | 2 (33.3%) |
| Surgeon | 4 (66.7%) | 4 (66.7%) |
| **Simulation experience (baseline), n (%)** |  |  |
