# Supplementary material for: Preliminary translational assessment of robotic surgery skills for vascular dissection: from simulator to in vivo porcine model
Source: J Robot Surg. 2026 Jul 20;20(1):730. doi: 10.1007/s11701-026-03662-5 (PMC13385068; doi:10.1007/s11701-026-03662-5)
Supplement: Supplementary file 3 — Supplementary Material 3 [file 11701_2026_3662_MOESM3_ESM.docx]

| Item | 1 | 2 | 3 | 4 | 5 | Trained | Not trained |
| --- | --- | --- | --- | --- | --- | --- | --- |
| **Depth Perception** | Consistently exceeds the target, large movements, fixes slowly. |  | Some failures in making the goal but corrected quickly. |  | Directs the instruments in the correct plane to the target | 5 | 2 |
| **Bimanual Dexterity** | Use of only one hand, ignores the non-dominant hand, poor coordination between the two. |  | Uses both hands, but the interaction between them is not optimal. |  | Use both hands in a complementary manner for optimal exposure | 4 | 3 |
| **Efficiency** | Many tentative movements, frequent changes in the things to do, no progress. |  | Slow movements, but organized and reasonable. |  | Confident and efficient, remains focused on the goal. | 4 | 2 |
| **Force Control** | Jerking, tearing the tissue, damage to structure. Frequent breaking of the suture. |  | Reasonable handling of tissues, less damage occurs. Occasional rupture of the suture. |  | Proper handling of tissues, proper traction thereof. Without breaking the suture. | 5 | 3 |
| **Autonomy** | Unable to complete the procedure. |  | The surgeon can complete the task safely, with some guidance tutor. | . | Able to complete the task alone, without a guide. | 5 | 5 |
| **Robot control** | No optimization of the position of the hands on the console, frequent collision. The vision is not optimal. |  | Occasional collision of instrument. Vision is sometimes not optimum |  | Adequate control of the camera. Optimal hand position without collision. . | 4 | 3 |
| **Total** |  |  |  |  |  | 27 | 18 |

**Appendix**

Case control 1

Case control 2

| Item | 1 | 2 | 3 | 4 | 5 | Trained | Not trained |
| --- | --- | --- | --- | --- | --- | --- | --- |
| **Depth Perception** | Consistently exceeds the target, large movements, fixes slowly. |  | Some failures in making the goal but corrected quickly. |  | Directs the instruments in the correct plane to the target | 4 | 2 |
| **Bimanual Dexterity** | Use of only one hand, ignores the non-dominant hand, poor coordination between the two. |  | Uses both hands, but the interaction between them is not optimal. |  | Use both hands in a complementary manner for optimal exposure | 5 | 3 |
| **Efficiency** | Many tentative movements, frequent changes in the things to do, no progress. |  | Slow movements, but organized and reasonable. |  | Confident and efficient, remains focused on the goal. | 5 | 4 |
| **Force Control** | Jerking, tearing the tissue, damage to structure. Frequent breaking of the suture. |  | Reasonable handling of tissues, less damage occurs. Occasional rupture of the suture. |  | Proper handling of tissues, proper traction thereof. Without breaking the suture. | 4 | 4 |
| **Autonomy** | Unable to complete the procedure. |  | The surgeon can complete the task safely, with some guidance tutor. | . | Able to complete the task alone, without a guide. | 4 | 3 |
| **Robot control** | No optimization of the position of the hands on the console, frequent collision. The vision is not optimal. |  | Occasional collision of instrument. Vision is sometimes not optimum |  | Adequate control of the camera. Optimal hand position without collision. . | 5 | 5 |
| **Total** |  |  |  |  |  | 27 | 21 |

Case control 3

| Item | 1 | 2 | 3 | 4 | 5 | Trained | Not trained |
| --- | --- | --- | --- | --- | --- | --- | --- |
| **Depth Perception** | Consistently exceeds the target, large movements, fixes slowly. |  | Some failures in making the goal but corrected quickly. |  | Directs the instruments in the correct plane to the target | 5 | 3 |
| **Bimanual Dexterity** | Use of only one hand, ignores the non-dominant hand, poor coordination between the two. |  | Uses both hands, but the interaction between them is not optimal. |  | Use both hands in a complementary manner for optimal exposure | 5 | 4 |
| **Efficiency** | Many tentative movements, frequent changes in the things to do, no progress. |  | Slow movements, but organized and reasonable. |  | Confident and efficient, remains focused on the goal. | 5 | 4 |
| **Force Control** | Jerking, tearing the tissue, damage to structure. Frequent breaking of the suture. |  | Reasonable handling of tissues, less damage occurs. Occasional rupture of the suture. |  | Proper handling of tissues, proper traction thereof. Without breaking the suture. | 4 | 4 |
| **Autonomy** | Unable to complete the procedure. |  | The surgeon can complete the task safely, with some guidance tutor. | . | Able to complete the task alone, without a guide. | 4 | 4 |
| **Robot control** | No optimization of the position of the hands on the console, frequent collision. The vision is not optimal. |  | Occasional collision of instrument. Vision is sometimes not optimum |  | Adequate control of the camera. Optimal hand position without collision. . | 4 | 3 |
| **Total** |  |  |  |  |  | 27 | 22 |

Case control 4

| Item | 1 | 2 | 3 | 4 | 5 | Trained | Not trained |
| --- | --- | --- | --- | --- | --- | --- | --- |
| **Depth Perception** | Consistently exceeds the target, large movements, fixes slowly. |  | Some failures in making the goal but corrected quickly. |  | Directs the instruments in the correct plane to the target | 5 | 4 |
| **Bimanual Dexterity** | Use of only one hand, ignores the non-dominant hand, poor coordination between the two. |  | Uses both hands, but the interaction between them is not optimal. |  | Use both hands in a complementary manner for optimal exposure | 5 | 4 |
| **Efficiency** | Many tentative movements, frequent changes in the things to do, no progress. |  | Slow movements, but organized and reasonable. |  | Confident and efficient, remains focused on the goal. | 5 | 4 |
| **Force Control** | Jerking, tearing the tissue, damage to structure. Frequent breaking of the suture. |  | Reasonable handling of tissues, less damage occurs. Occasional rupture of the suture. |  | Proper handling of tissues, proper traction thereof. Without breaking the suture. | 4 | 4 |
| **Autonomy** | Unable to complete the procedure. |  | The surgeon can complete the task safely, with some guidance tutor. | . | Able to complete the task alone, without a guide. | 4 | 4 |
| **Robot control** | No optimization of the position of the hands on the console, frequent collision. The vision is not optimal. |  | Occasional collision of instrument. Vision is sometimes not optimum |  | Adequate control of the camera. Optimal hand position without collision. . | 5 | 4 |
| **Total** |  |  |  |  |  | 28 | 24 |

Case control 5

| Item | 1 | 2 | 3 | 4 | 5 | Trained | Not trained |
| --- | --- | --- | --- | --- | --- | --- | --- |
| **Depth Perception** | Consistently exceeds the target, large movements, fixes slowly. |  | Some failures in making the goal but corrected quickly. |  | Directs the instruments in the correct plane to the target | 4 | 3 |
| **Bimanual Dexterity** | Use of only one hand, ignores the non-dominant hand, poor coordination between the two. |  | Uses both hands, but the interaction between them is not optimal. |  | Use both hands in a complementary manner for optimal exposure | 4 | 3 |
| **Efficiency** | Many tentative movements, frequent changes in the things to do, no progress. |  | Slow movements, but organized and reasonable. |  | Confident and efficient, remains focused on the goal. | 4 | 3 |
| **Force Control** | Jerking, tearing the tissue, damage to structure. Frequent breaking of the suture. |  | Reasonable handling of tissues, less damage occurs. Occasional rupture of the suture. |  | Proper handling of tissues, proper traction thereof. Without breaking the suture. | 4 | 3 |
| **Autonomy** | Unable to complete the procedure. |  | The surgeon can complete the task safely, with some guidance tutor. | . | Able to complete the task alone, without a guide. | 4 | 4 |
| **Robot control** | No optimization of the position of the hands on the console, frequent collision. The vision is not optimal. |  | Occasional collision of instrument. Vision is sometimes not optimum |  | Adequate control of the camera. Optimal hand position without collision. . | 5 | 4 |
| **Total** |  |  |  |  |  | 25 | 20 |

Case control 6

| Item | 1 | 2 | 3 | 4 | 5 | Trained | Not trained |
| --- | --- | --- | --- | --- | --- | --- | --- |
| **Depth Perception** | Consistently exceeds the target, large movements, fixes slowly. |  | Some failures in making the goal but corrected quickly. |  | Directs the instruments in the correct plane to the target | 3 | 3 |
| **Bimanual Dexterity** | Use of only one hand, ignores the non-dominant hand, poor coordination between the two. |  | Uses both hands, but the interaction between them is not optimal. |  | Use both hands in a complementary manner for optimal exposure | 3 | 3 |
| **Efficiency** | Many tentative movements, frequent changes in the things to do, no progress. |  | Slow movements, but organized and reasonable. |  | Confident and efficient, remains focused on the goal. | 3 | 3 |
| **Force Control** | Jerking, tearing the tissue, damage to structure. Frequent breaking of the suture. |  | Reasonable handling of tissues, less damage occurs. Occasional rupture of the suture. |  | Proper handling of tissues, proper traction thereof. Without breaking the suture. | 4 | 3 |
| **Autonomy** | Unable to complete the procedure. |  | The surgeon can complete the task safely, with some guidance tutor. | . | Able to complete the task alone, without a guide. | 3 | 3 |
| **Robot control** | No optimization of the position of the hands on the console, frequent collision. The vision is not optimal. |  | Occasional collision of instrument. Vision is sometimes not optimum |  | Adequate control of the camera. Optimal hand position without collision. . | 4 | 5 |
| **Total** |  |  |  |  |  | 20 | 20 |
